# Heat Treatment Optimization for a High Strength Al–Mn–Sc Alloy Fabricated by Selective Laser Melting

**DOI:** 10.3390/ma16114054

**Published:** 2023-05-29

**Authors:** Hongyu Liu, Hao Zhang, Liju Meng, Yulong Li, Sheng Cao

**Affiliations:** 1 Department of Mechanical Engineering, College of Engineering, Shantou University, Shantou 515063, China; 2 Faculty of Materials and Manufacturing, Beijing University of Technology, Beijing 100080, China

**Keywords:** Al–Mn–Sc alloy, selective laser melting, heat treatment, high strength, precipitation hardening

## Abstract

A selective laser-melted Al–Mn–Sc alloy with 99.9% relative density has been obtained in this work through systematic process optimization. The as-fabricated specimen had the lowest hardness and strength, but the highest ductility. The aging response has shown that 300 °C/5 h is the peak aged condition, and it had the highest hardness, yield strength, ultimate tensile strength, and elongation at fracture. Such a high strength was attributed to the uniformly distributed nano-sized secondary Al_3_Sc precipitates. A further increase in aging temperature to 400 °C resulted in an over-aged condition, which contained a reduced volume fraction of secondary Al_3_Sc precipitates and resulted in a reduced strength.

## 1. Introduction

Selective laser melting (SLM), as a widely employed additive manufacturing (AM) technology, is an emerging technology in the energy, aerospace, and medical fields [1,2]. Compared to conventional manufacturing techniques of casting [3], rolling [4], forging [5], and extrusion [6], SLM utilizes its flexible manufacturing characteristics to directly fabricate geometrically complex parts, layer by layer, through precise control of a high-energy laser beam on the metallic powder bed. The highly localized melting, the ultra-fast cooling rate (10^5^–10^6^ K/s), and the extremely high temperature gradient (G–10^6^ K/m) allow the formation of extraordinary non-equilibrium microstructures and excellent mechanical properties in SLM manufactured parts [7,8]. Furthermore, the unutilized powder in the preparation process can be recycled and reused, which effectively reduces the usage and wastage of raw materials [9].

SLM fabricated aluminum (Al) alloys are known for their excellent strength–to–weight ratio and good corrosion resistance. The 2xxx, 6xxx, and 7xxx series high-strength Al alloys realize high specific strengths which are comparable to high-strength steels [10]. However, it is very challenging to use SLM to fabricate high-strength commercial aluminum alloy parts with large dimensions due to their high solidification cracking (hot tearing) susceptibility [2,11]. Therefore, to avoid solidification cracks and to improve the SLM processibility, it is significant to extend to applications of SLM fabricated high-strength Al alloys.

To reduce and eliminate the crack susceptibility of SLM fabricated high-strength aluminum alloys, the inclusion of Sc microalloying elements was effective in previous studies [12,13,14]. The addition of Sc elements facilitates the formation of fine equiaxed grains, which can effectively accommodate the strain generated during SLM, and avoid solidification cracking. Other studies found that post-heat treatment triggered the precipitation of Sc enriched nano-particles and resulted in a high volume fraction of secondary Al_3_Sc nano-precipitates, which can effectively improve the strength [15]. Jia et al. [16,17] designed a high-strength Al–Mn–Sc alloy with a tensile strength of 560 MPa and a fracture strain of 18%. Such a high strength was achieved by contributions of a grain refining effect by the addition of Sc, solid solution strengthening by the supersaturate Mn, and precipitation hardening by the formation of secondary Al_3_Sc nanoprecipitates [18,19]. All the above studies focused on the as-fabricated and post-process aged conditions (aging temperature generally at 300 °C); however, only a few investigations have been conducted to investigate the effect of different heat treatment temperatures and times on the room temperature mechanical properties of SLM fabricated Al–Mn–Sc alloys.

In this paper, the SLM process optimization has been carried out for the Al–Mn–Sc alloy. In addition, the influences of heat treatment parameters on the room temperature mechanical properties of a SLM fabricated high-strength Al–Mn–Sc alloy have been investigated. The microstructure evolution has been systematically investigated by scanning electron microscope (SEM), electron-backscattered diffraction (EBSD), and transmission electron microscope (TEM). The room temperature mechanical properties have been studied in terms of room temperature hardness and tensile tests.

## 2. Materials and Methods

### 2.1. Material, SLM Fabrication, and Post-Process Heat Treatment

**Figure 1:**
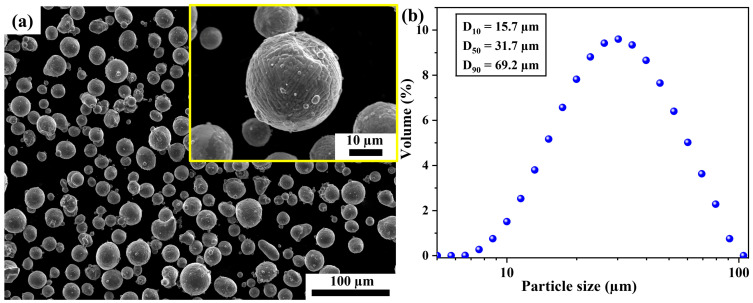
The particle characteristics of Al–Mn–Sc powders. (**a**) A SEM secondary electron (SE) image of the Al–Mn–Sc alloy powder and (**b**) particle size distribution of the pre-alloy powders. The yellow square in (**a**) is an enlarged view for the powder particle.

Argon gas atomized powders with a nominal composition of Al-1.5~3.5%Mn-1.5~2%Mg-0.5~1%Sc-0.2~0.3%Zr (wt.%) were provided by Material Technology Innovations Co., Ltd., (Guangzhou, China). Figure 1a shows that the particle morphology of the Al–Mn–Sc alloy powder is generally spherical. Figure 1b describes the powder particle size distribution determined by laser diffraction by using a Mastersizer 2000 device (Malvern panalytical ltd., UK). The pre-alloyed Al–Mn–Sc powders had the D(10), D(50), and D(90) at 15.7 μm, 31.7 μm, and 69.2 μm, respectively.

The SLM samples were fabricated by using an AmPro SP260 machine (AMPro Innovations Co., Ltd., China) equipped with a 500 W laser source. These specimens were built on an Al substrate. To obtain an SLM processing window, laser power from 200 to 370 W and scan speed from 800 to 2000 mm/s had been systematically investigated. The substrate was preheated to 100 °C. In addition, a hatch distance of 0.14 mm and a layer thickness of 0.03 mm were selected in the SLM fabrication process. For all specimens, a strip scan strategy with a scanning rotation of 67° between the consecutive layers was used.

After SLM fabrication, the post-process heat treatments at different temperatures from 250 to 400 °C and different times up to 24 h were performed. The heat treatment experiment was carried out in an air furnace equipped with a K-type thermocouple. After the post-process heat treatment, the skin layer of the specimens was either machined or ground with SiC sandpaper to avoid the oxidation influence on the following microstructure characterization and mechanical property evaluation.

### 2.2. Microstructural Observation

The samples for microstructural and hardness characterization were ground to a 5000-grit finish using SiC sandpapers, and were then polished using a 50 nm SiO_2_ Nano-MAX suspension. The metallographic samples were then etched by a Keller solution for 15 to 30 s. The optical micrograph (OM) was obtained by using a ZEISS Axioskop microscope (LSM800, Zeiss, Germany). A DH-200M density instrument was used to measure the relative density of the samples according to Archimedes’ principle. SEM images were examined using a JSM-7200F microscope (JEOL, Ltd., Japan), and inverse pole figure (IPF), and grain size distribution maps were obtained by using the same SEM equipped with an EDAX Velocity Super electron back-scattered diffraction (EBSD) system (EDAX Inc., USA). TEM and scanning TEM (STEM) specimens with a diameter of 3 mm were mechanically ground to a thickness of 130 μm and then ion polished by using a Gatan 691 system at an angle of 5° and a voltage of 3.6 KeV. TEM and STEM experiments were carried out to investigate the small-sized precipitates by using a FEI Tecnai F30 microscope (Thermo Fisher Scientific Inc., USA) operating at 200 KV. The size of these precipitates was measured by Image-Pro Plus 6.0 software.

### 2.3. Mechanical Test

The hardness test was performed to evaluate the aging response for LPBF fabricated specimens aged at different temperatures and durations. The Vickers hardness was measured by using a HXD-1000TM (Shanghai Taiming Optical Instrument Co., Ltd., China) with tester at a constant load of 300 g and a holding time of 10 s, and 10 indentations were measured to obtain an average hardness value for each sample. A room temperature tensile test was carried out on a GNT-50 machine (NCS testing technology Co., Ltd., China) with a constant cross-head moving speed of 1 mm/min and a 10 mm extensometer. Three replicates were used to determine the average tensile properties.

## 3. Results

### 3.1. Relative Density

**Figure 2:**
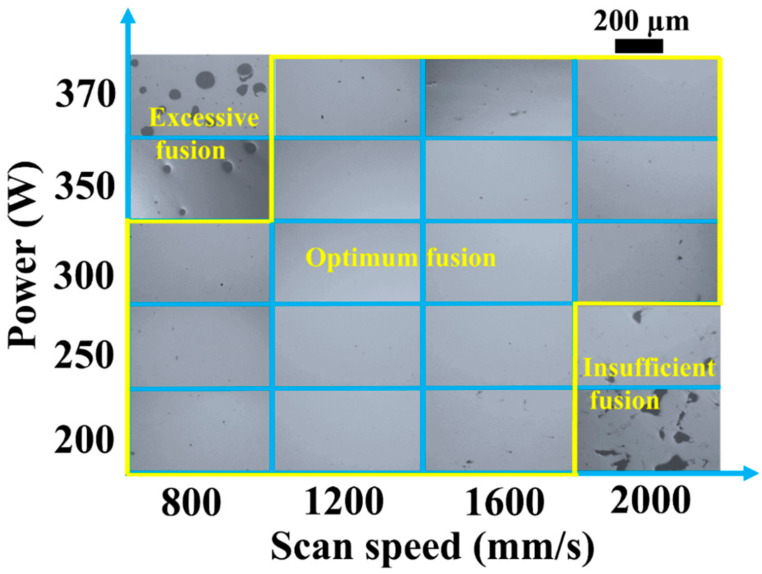
Optical micrographs of SLM fabricated Al–Mn–Sc alloys built at different laser parameters.

Figure 2 shows representative OM images of Al–Mn–Sc specimens built at different LPBF parameters. Irregular lack-of-fusion defects were observed at low laser powers and high scan speeds due to insufficient energy input [20], while some keyholes’ defects appeared at high laser powers and low scan speeds as a result of excessive laser energy [21]. An optimum process window was located at a parameter range between these two regions, and specimens with high relative densities were obtained. The relative densities of these specimens were then measured by the Archimedes method. The specimen with the highest relative density of 99.9 ± 0.1% was built at a laser power of 350 W and a scanning speed of 1200 mm/s, and these laser parameters were employed for the specimen used in the following microstructural and mechanical investigations.

### 3.2. The Microstructure Characteristics and Aging Response

**Figure 3:**
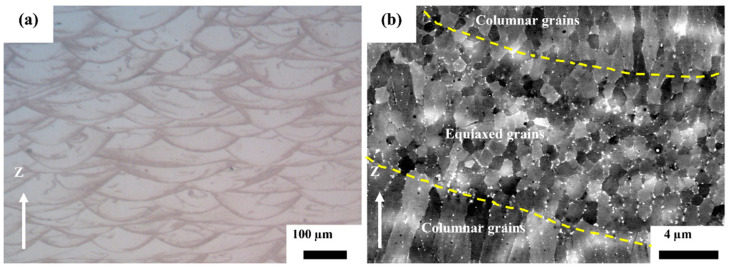
The microstructural characteristics of the SLM fabricated Al–Mn–Sc alloy. (**a**) OM image confirms a high relative density and (**b**) BSE image shows the heterogeneous grain structure.

Figure 3 shows the microstructural characteristics of the as-fabricated Al–Mn–Sc specimen with the highest relative density. The specimen was generally fully dense with only a few small defects in dark contrast as shown in Figure 3a. Figure 3b shows the as-fabricated microstructure having a heterogeneous microstructure of columnar grains (CG) within melt pool and equiaxed grains (EG) at the melt pool boundary. In addition, there were some bright intergranular and intragranular particles in both the CG and EG regions.

**Figure 4:**
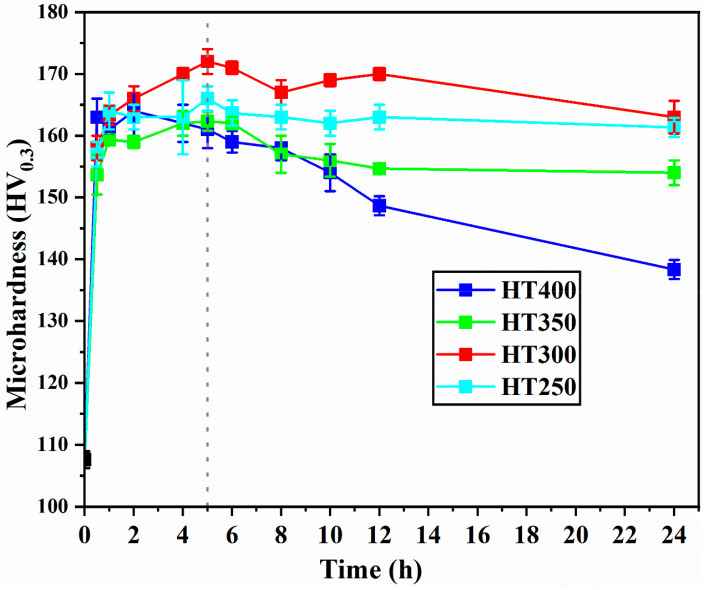
Hardness of the SLM fabricated and post-process heat-treated Al–Mn–Sc alloy aged at different temperatures. Errors are the standard deviations, and the black dotted line indicates the peak hardness was achieved at 5 h for 300 °C.

Figure 4 illustrates the hardness evolution at different aging temperatures for the SLM fabricated Al–Mn–Sc alloy. It shows that the heat treatment temperature and heat treatment time had significant effects on the aging response. The hardness of the as-fabricated specimen was approximately at 108 HV_0.3_. It increased significantly when the specimen was subjected to aging treatment at all temperatures. The peak hardness was the highest at 172 HV_0.3_ for the aged specimen at 300 °C, and it was then followed by 166 HV_0.3_ at 250 °C, 164 HV_0.3_ at 350 °C, and 162 HV_0.3_ at 400 °C. Additionally, the time required to reach peak hardness decreased with the increased heat treatment temperatures. For instance, it took 5 h to reach the peak hardness at 250 °C. In comparison, only 2 h was required at 400 °C to reach the peak hardness. This trend agrees with the general rule that precipitation kinetics accelerate at high heat treatment temperatures [22]. Moreover, it remained at a stable hardness up to 24 h when low aging temperatures of 250 and 300 °C were used. In contrast, the hardness value dropped quickly from the peak hardness at high aging temperatures of 350 and 400 °C. This observation indicates the good thermal stability of the SLM fabricated Al–Mn–Sc alloy at temperatures up to 300 °C.

**Figure 5:**
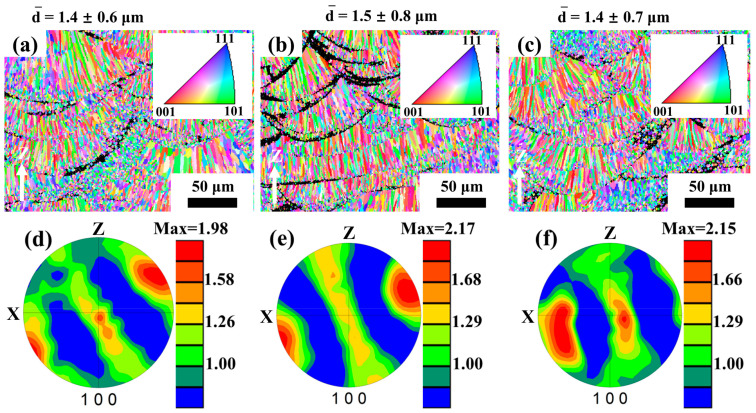
The EBSD-IPF maps, average grain size, and their corresponding pole figures of the SLM fabricated and post-process heat-treated Al–Mn–Sc alloy. (**a**,**d**) As-fabricated specimen; (**b**,**e**) aged at 300 °C for 5 h; and (**c**,**f**) aged at 400 °C for 5 h.

To further investigate the microstructure and tensile properties, the as-fabricated specimen, a peak aged condition at 300 °C for 5 h (HT300), and an over aged condition at 400 °C for 5 h (HT400) were chosen in the following study. Figure 5 shows the IPF maps and associated grain size distribution of the specimen aged at as-fabricated, and post-process heat-treated specimens at 300 and 400 °C for 5 h. The IPF map in Figure 5a confirms the existence of the heterogeneous microstructure of CG and EG in the selective laser-melted Al–Mn–Sc specimen. It had an average grain size of α-Al at 1.4 ± 0.6 µm. Similar to the as-fabricated condition, the aged samples also exhibited heterogeneous microstructures of equiaxed grains and columnar grains. The average grain size of the two specimens was 1.5 ± 0.8 μm for 300 °C/5 h and 1.4 ± 0.7 μm for 400 °C/5 h, respectively. The α-Al grain sizes were generally similar for the as-fabricated specimens and these two post-process aged specimens. Furthermore, Figure 5d–f show the (100) pole figures corresponding to Figure 5a–c. For all specimens, the (100) texture components are weak at ~2, which indicates that heat treatment has no significant effect on the texture evolution in the SLM fabricated Al–Mn–Sc alloy. This texture result is consistent with previous studies [16,23,24].

**Figure 6:**
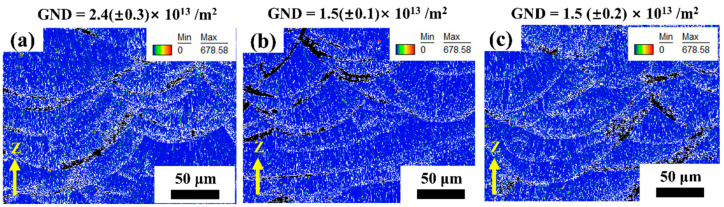
The GND densities of as-fabricated and post-process aged Al–Mn–Sc alloy samples calculated from EBSD data. (**a**) As-fabricated specimen; (**b**) the post-process aged specimen at 300 °C for 5 h; and (**c**) the post-process aged specimen at 400 °C for 5 h.

The EBSD data were then analyzed to obtain geometrically necessary dislocation (GND) distribution in these samples. Figure 6 depicts the GND densities of the Al–Mn–Sc alloy in both as-fabricated and aged states. The GND distribution revealed a higher dislocation density in equiaxed grains at the melt pool boundary. A previous study showed that large columnar grains generally had a low GND density [25]. To further quantify the degree of deformation, the geometrically necessary dislocation (GND) density was calculated based on the GND maps [26]. Among the three specimens, GND density was the highest at 2.4 (±0.3) × 10^13^/m^2^ in the as-fabricated specimens, and it decreased to 1.5 (±0.1) × 10^13^/m^2^ and 1.5 (±0.2) × 10^13^/m^2^ for the aged specimens of 300 and 400 °C, respectively.

**Figure 7:**
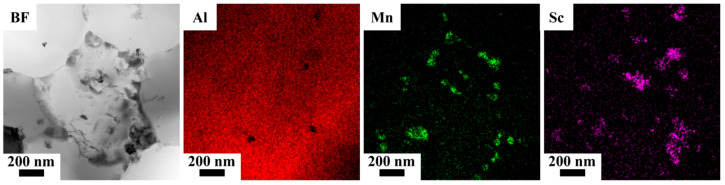
STEM bright field (BF) image and associated EDS element maps of the as-fabricated Al–Mn–Sc sample.

To study those bright particles in Figure 3b, a STEM-EDS experiment was performed. As shown in Figure 7, there were Mn enriched and Sc enriched particles. The Mn enriched particle should be Al_6_Mn precipitates, and the Sc enriched particle should be primary Al_3_Sc precipitates. Al_6_Mn and primary Al_3_Sc precipitates have been widely reported in selective laser-melted Al–Mn–Sc alloys [9].

**Figure 8:**
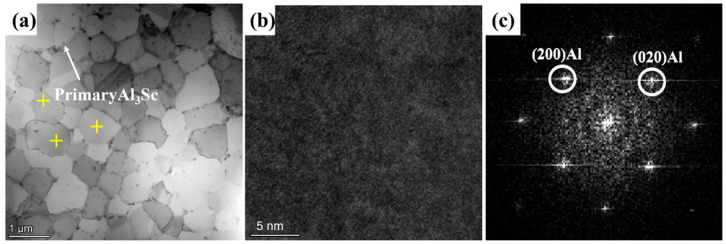
TEM characterization of as-fabricated sample. (**a**) A bright field (BF) image; (**b**) a high resolution TEM image in the equiaxed grain; and (**c**) a [001] fast Fourier transformation (FFT) from image (**b**). Yellow crosses are the locations for EDS point analyses.

Figure 8a presents equiaxed grain regions by a TEM bright-field (BF) image of a selective laser-melted Al–Mn–Sc specimen in the as-fabricated condition. In addition to the equiaxed grains, there were some Al_6_Mn and primary Al_3_Sc precipitates. Figure 8b is a high resolution TEM image of an equiaxed grain. The associated [001] fast Fourier transformation (FFT) in Figure 8c indicates that there was only α-Al matrix.

**Figure 9:**
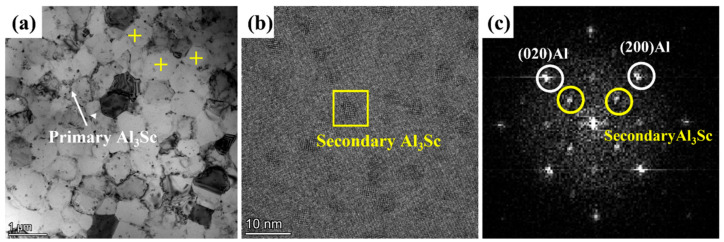
TEM characterization of the post-process aged sample at 300 °C (HT300). (**a**) A bright field (BF) image; (**b**) a high resolution TEM image; and (**c**) a [001] FFT from image (**b**). Yellow crosses are the locations for EDS point analyses.

Figure 9 shows a TEM BF image, an HRTEM image, and the associated FFT of the post-process heat-treated Al–Mn–Sc specimen aged at 300 °C for 5 h (HT300). As shown in Figure 9a, the equiaxed grain size after aging treatment was similar to that of the as-built condition, and there were some Al_6_Mn and primary Al_3_Sc precipitates. HRTEM and associated [001] FFT in Figure 9b,c revealed the existence of secondary Al_3_Sc nano-precipitates with a size of ~3.05 ± 0.54 nm according to the superlattice reflections. The precipitation of secondary Al_3_Sc has been reported in SLM fabricated and heat-treated Sc containing Al alloys [9,24,27].

**Figure 10:**
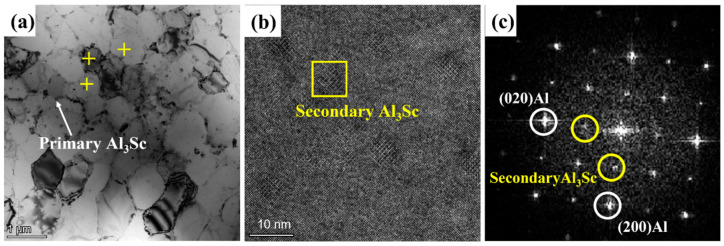
TEM characterization of the post-process aged sample at 400 °C (HT400). (**a**) A bright field (BF) image; (**b**) a high resolution TEM image; and (**c**) a [001] FFT from image (**b**). Yellow crosses are the locations for EDS point analyses.

Figure 10 illustrates a TEM BF image, an HRTEM image, and associated FFT of the post-process heat-treated Al–Mn–Sc specimen aged at 400 °C for 5 h (HT400). In general, the equiaxed grain region, Al_6_Mn, and primary Al_3_Sc precipitates were similar to the as-fabricated and HT300 specimens. In addition, secondary Al_3_Sc nano-precipitates were observed in the α-Al matrix, and their size (~5.51 ± 0.72 nm) seemed to be larger than those in the HT300 specimens. In addition, the number density of secondary Al_3_Sc was reduced in the HT400 specimen compared to that in the HT300 specimen.

### 3.3. Tensile Test

**Figure 11:**
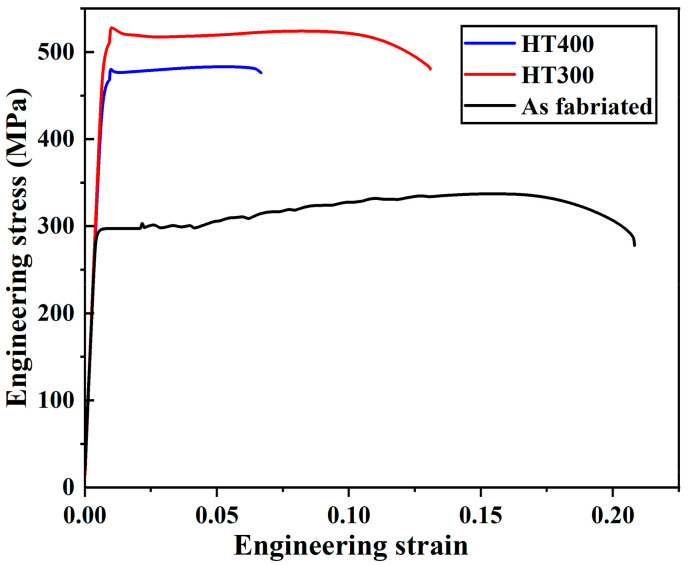
The representative engineering stress–strain curves obtained in uniaxial tensile tests for SLM fabricated and post-process aged specimens.

Three replicates were tested in uniaxial tensile loading for SLM fabricated and post-process aged Al–Mn–Sc specimens, and the representative stress–strain curves are illustrated in Figure 11. The average yield strength (YS), ultimate tensile strength (UTS), and uniform elongation at failure (El.) are summarized in Table 1. The as-fabricated specimen had the lowest YS at ~296 MPa and the highest El. at 18.8%. Aging treatments effectively improved both the YS and UTS but decreased the ductility. The peak aged condition of HT300 possessed a YS of ~502 MPa and an El. of 13.1%. Once the specimen was overaged (HT400), both the YS and El. decreased.

## 4. Discussion

The hardness evolutions and strength curves shown in Figure 4 and Figure 11 demonstrate that the SLM fabricated Al–Mn–Sc alloy can achieve good mechanical performance through post-process heat treatment. The main contributions to yield strength are: (i) grain size strengthening; (ii) solid solution strengthening; (iii) dislocation strengthening; and (iv) precipitation strengthening by secondary nano-sized Al_3_Sc.

According to the Hall–Petch relationship [28], given by Equation (1):

(1) $$\sigma_{GS}=\sigma_{0}+k_{d}d_{mean}{}^{-0.5}$$

where *σ*_0_ is the friction stress of pure aluminum (about 20 MPa, *k_d_* is the Hall–Petch strengthening coefficient (0.17 MPa m^0.5^), and *d_mean_* is the average grain size shown in Figure 5. The contribution from grain size strengthening is, thereby, calculated at 158, 164, and 158 MPa for as-fabricated, HT300, and HT400 samples, respectively.

For solid solution strengthening, the content of Mn and Mg in the Al–Mn–Sc alloy is the main source for solid solution strengthening [16]. The yield strength increase due to the solid solution can be estimated using the following equation [29]:

(2) $$\sigma_{ss}=HC^{n}$$

where *H* is a strengthening coefficient and *n* is a concentration exponent, and their values can be found in Ref. [18]. *C* is the concentration of the solute in atomic percentage in Table 2. According to this formula, the solid solution strengthening contribution is 110, 116, and 115 MPa for as-fabricated, HT300, and HT400, respectively. The Sc generally forms primary and secondary Al_3_Sc precipitates, and the Sc in solid solution is low at less than 0.2 at.%. As a result, Sc has a negligible contribution on the solid solution strengthening effect.

As demonstrated in Figure 6, the GND densities for all specimens were low at the order of 10^13^, and this is consistent with a previous study [30]. After the aging treatment, the GND density was slightly decreased from ~2.4 × 10^13^ m^−2^ in the as-fabricated specimen to ~1.5 × 10^13^ m^−2^ in the HT300 and HT400 specimens. The dislocation strengthening can be assessed by the Bailey–Hirsch relationship [31]:

(3) $$\sigma_{DS}=M\alpha Gb\rho^{0.5}$$

where *M* is the Taylor factor (3.06 for FCC crystals [32]); α is constant (*α* = 0.24 [32]); *G* is the shear modulus (25.4 GPa [32]); *b* is the Burgers vector (*b* = 0.286 nm [32]); and *ρ* is the GND, as shown in Figure 6. The dislocation strengthening was estimated at ~26 MPa for the as-fabricated Al–Mn–Sc sample and ~21 MPa for both the HT300 and HT400 specimens. Therefore, the dislocation strengthening is not the main cause for the strength difference among the as-fabricated and aged specimens, as shown in Figure 11.

In the SLM fabricated Al–Mn–Sc alloys, the precipitation hardening is generally attributed to secondary Al_3_Sc nano-precipitates rather than the large-sized Al_6_Mn and primary Al_3_Sc precipitates [15]. The secondary Al_3_Sc precipitates had a larger size at 5.51 ± 0.72 nm in the HT400 specimen than that of 3.05 ± 0.54 nm in the HT300 specimen. This suggests that higher aging temperature significantly coarsens the secondary Al_3_Sc precipitates [33]. For precipitates with size below 8 nm (Figure 9b and Figure 10b), they are sheared by dislocations rather than bypassed through the Orowan dislocation looping mechanism during deformation [18,34,35]. Considering that the secondary Al_3_Sc precipitates in both the HT300 and HT400 specimens were smaller than 8nm, the precipitation hardening effect can be evaluated by the precipitate shearing mechanism [36]. The strengthening contribution from precipitate hardening can be evaluated by the Nembach’s equation [37]:

(4) $$\sigma_{PS}=0.0055M{\left(\Delta G\right)}^{\frac{3}{2}}\;{\left(\frac{2f_{v}}{G}\right)}^{\frac{1}{2}}\;{\left(\frac{r}{b}\right)}^{\left(\frac{3}{2}m-1\right)}$$

where *M* = 3.06 [38]; *G* = 25.4 GPa is the shear modulus of Al [38]; *r* is the average precipitates radius; *b* = 0.286 nm is the matrix Burgers vector; Δ*G* = 42.6 GPa is the shear modulus difference between the Al matrix and secondary Al_3_Sc precipitates [39]; *m* is a constant at 0.85; and *f_v_* is the volume fraction of precipitates (1.22 Vol.% and 0.6 Vol.% for HT300 and HT400 specimens, respectively). The *σ_ps_* were calculated at 227 and 185 MPa for 300 T and 400 T. The high volume fraction of extremely fine-sized secondary Al_3_Sc precipitates in HT300 would lead to a stronger precipitation strengthening effect than that in HT400.

Overall, considering the grain size strengthening, solid solution strengthening, dislocation strengthening, and precipitation strengthening, the estimated yield strength can be determined as:

(5) $$\sigma_{0.2-estimate}=\sigma_{GS}+\sigma_{ss}+\sigma_{DS}+\sigma_{PS}$$

Table 3 shows the contributions from individual strengthening mechanisms and the overall estimated yield strengths for the as-fabricated, HT300 and HT400 specimens. The yield strength estimation is consistent with the experimental results. Among these three specimens, there were no apparent differences in terms of the contributions from the solid solution strengthening, the grain boundary strengthening, and the dislocation strengthening. The yield strength differences among these three specimens were from the precipitation strengthening. The uniform distributed fine secondary Al_3_Sc nano-precipitates significantly increased the yield strength from ~296 MPa in the as-fabricated specimen to ~502 MPa in the peak aged condition of HT300, and the overaged HT400 had a slightly decreased yield strength to ~464 MPa due to the reduced volume fraction of secondary Al_3_Sc precipitates as discussed in the previous paragraph.

## 5. Conclusions

This work systematically investigated the influence of laser parameters on the relative density of a selective laser-melted Al–Mn–Sc alloy. In addition, the impact of post-process aging treatment on the microstructure and mechanical properties has been studied. The main conclusions are summarized as follows:

(1) An optimized SLM parameter set was obtained at a laser power of 350 W, a scan speed of 1200 mm/s, a hatch distance of 140 μm, and a layer thickness of 30 μm for the Al–Mn–Sc alloy with 99.9% relative density. The as-fabricated specimen had the lowest hardness, yield strength, and ultimate tensile strength, but it had the highest elongation at fracture.
(2) The aging response showed that 300 °C for 5 h is the peak aged condition for the SLM fabricated Al–Mn–Sc alloy. A high yield strength of ~502 MPa was obtained in the peak aged condition (HT300) due to the uniformly distributed nano-sized secondary Al_3_Sc precipitates, and peak aged condition still had a good elongation at fracture of 13.1%.
(3) The strength decreased in the overaged conditions of HT400, which was attributed to the reduced volume fraction of secondary Al_3_Sc precipitates at a high aging temperature.

## Figures and Tables

**Table 1:** The average yield strengths (YS), ultimate tensile strength (UTS), and uniform elongation at failure (EI.) for SLM fabricated and post-process aged specimens. Three replicates were tested for each condition, and errors represented the standard deviations.

Specimen Condition	YS (MPa)	UTS (MPa)	El. (%)
As-fabricated	296 ± 2	337 ± 3	18.8 ± 3.1
HT300	502 ± 4	527 ± 4	13.1 ± 2.7
HT400	464 ± 4	483 ± 4	6.2 ± 1.0

**Table 2:** STEM-EDS data of Al matrix (EDS collection locations are highlighted by the yellow crosses) from Figure 8a, Figure 9a and Figure 10a (at.%).

Specimen Condition	Mn	Mg	Sc	Zr
As-fabricated	1.7 ± 0.4	1.4 ± 0.2	0.1 ± 0.1	0.1 ± 0.1
HT300	1.8 ± 0.3	1.4 ± 0.4	0.2 ± 0.1	0.2 ± 0.1
HT400	1.8 ± 0.2	1.3 ± 0.3	0.2 ± 0.1	0.2 ± 0.2

**Table 3:** Calculated strength contribution from different strengthening mechanisms before and after heat treatment compared with the experimental tensile yield strength.

Strengthening Contribution	As-Fabricated	300HT	400HT
*σ_GS_* (MPa)	158	164	158
*σ_ss_* (MPa)	110	116	115
*σ_DS_* (MPa)	26	21	21
*σ_PS_* (MPa)	0	227	185
*σ*_0.2-*estimated*_ (MPa)	294	528	479
*σ*_0.2-*experimental*_ (MPa)	296	502	464

## Data Availability

Data will be made available on request.
